# Therapeutic effects of topical 5-aminolevulinic acid photodynamic therapy

**DOI:** 10.12669/pjms.324.9634

**Published:** 2016

**Authors:** Yin-E Hu, Shu-Fang Dai, Bin Wang, Wei Qu, Jun-Ling Gao

**Affiliations:** 1 Yin-E Hu, Department of Dermatology, Huaihe Hospital of Henan University, Kaifeng 475000, Henan Province, China; 2 Shu-Fang Dai, Department of Dermatology, Huaihe Hospital of Henan University, Kaifeng 475000, Henan Province, China; 3 Bin Wang, Department of Dermatology, Huaihe Hospital of Henan University, Kaifeng 475000, Henan Province, China; 4 Wei Qu, Department of Dermatology, Huaihe Hospital of Henan University, Kaifeng 475000, Henan Province, China; 5 Jun-Ling Gao, Department of Dermatology, Huaihe Hospital of Henan University, Kaifeng 475000, Henan Province, China

**Keywords:** 5-aminolevulinic acid, Clinical therapeutic effect, Genital wart, Photodynamic therapy, Safety

## Abstract

**Objective:**

To evaluate the therapeutic effects of combined 5-aminolevulinic acid (ALA) and photodynamic therapy (PDT) on genital warts and the safety.

**Methods:**

One hundred ten patients with genital warts who were treated in our hospital from June 2013 to October 2014 were selected. The warts and affected parts were disinfected with benzalkonium bromide solution, and the warts were covered with absorbent cotton that had already been added freshly prepared 20% ALA solution, packaged and fixed. Then they were wet-dressed in dark, into which ALA solution was added according to the proportion of 5:3:2 every 30 minutes for three consecutive hours. Afterwards, the warts were illuminated by using photodynamic laser apparatus. The clinical outcomes, adverse reactions and recurrence rates were observed.

**Results:**

Genital warts were relieved in 107 out of the 110 cases (cure rate: 97.3%). Male patients had significantly better treatment outcomes at the urethral orifice than those in other affected parts. In the 107 patients, the cure rate of male patients was 98.8%, and they were cured after being treated four times. In contrast, female patients, who were cured after 5 times of treatment, had the cure rate of 91.7%. Their cure rates were similar (χ^2^=0, P>0.05), but the males were cured after significantly fewer times of treatment than the females (t=-7.432, P<0.05). Five patients suffered from mild tingling or burning sensation upon dressing at the urethral orifice, and the others were all free from systemic adverse reactions. After illumination, a small portion of the patients had mildly red, swelling, painful affected parts, with mild edema that almost disappeared within three days. Three patients relapsed at the urethral orifice and were then cured after further treatment.

**Conclusion:**

ALA-PDT can treat genital warts safely with high cure rate and low recurrence rate, particularly working for those of males at the urethral orifice.

## INTRODUCTION

Genital wart is a common sexually transmitted disease induced by human papillomavirus (HPV) infections of the genitalia, anus or perineum.^1^ This disease has high incidence and recurrence rates. Traditionally, genital warts are treated by surgical methods such as laser, electrocautery and freezing in combination with smearing of cytotoxic drugs, which, however, only eliminate those on the surface and work badly for subclinical infections. Besides, they usually induce adverse reactions such as local ulcers, erosions and even bleeding, for which combined 5-aminolevulinic acid (ALA) and photodynamic therapy (PDT) was developed. Tissues with pathological changes can selectively absorb and store photosensitizers that are activated to produce reactive oxygen species upon excitation at specific wavelengths. As a result, the species react with biomacromolecules in cells to trigger cytotoxic response (e.g. cells are subjected to morphological changes, injury or death) to cure the warts.^2^,^3^ In this study, genital warts of the external genitalia were treated by ALA-PDT, showing satisfactory outcomes.

## METHODS

### General information

A total of 110 patients with genital warts who were treated in our hospital from June 2013 to October 2014 were selected, comprising 84 males and 26 females aged from 18 to 66 years old (mean: 30.4 ± 10.3). All patients received treatment for the first time. The disease courses ranged from one week to nine months, with the average of (4.2 ± 6.3) months. The patients had 148 affected parts: 47 at the urethral orifice, 23 at the penis body, 20 at the foreskin, 20 at the glans penis, 16 at the coronal sulcus, 12 at the frenulum, 8 at the labia majora and 2 at the vaginal orifice. The warts were sized 1~4 mm^3^.

### Inclusion criteria

Genital warts were diagnosed by the vinegar test. The patients with positive results, who had warts of the external genitalia for the first time, were included in this study. The largest warts had the diameters of ≤1 cm, and they were systematically treated for the first time.^3^ This study was approved by the ethics committee of our hospital, and written consent has been obtained from all patients.^4^

### Exclusion criteria

The patients with porphyria, light allergy, history of drug allergy, low immunity, coagulopathy or proneness to scar were excluded. In addition, pregnant and lactating women were also excluded.^5^

### Methods

The warts and affected parts were disinfected with benzalkonium bromide solution (1:1000 dilution), and the warts were covered with absorbent cotton (diameter: 1.0 cm) that had already been added freshly prepared 20% ALA solution (Shanghai Fudan-Zhangjiang Bio-Pharmaceutical Co., Ltd.), packaged with sterilized plastic film and fixed with desensitized tape. Then the warts were wet-dressed in dark while the patients took a rest, into which ALA solution was added according to the proportion of 5:3:2 every 30 minutes for three consecutive hours. Afterwards, they were illuminated by using a photodynamic laser apparatus (XD-635AB, Guilin Xingda Photoelectric Co., Ltd.). The energy density of illumination was regulated between 100 and 150 J/cm^2^ according to age, degree of injury and degree of tolerance.^6^,^7^

The patients were followed up and re-examined one week after treatment to record changes in skin damages and regression time periods. Adverse reactions such as ulcers, erosions, redness, swelling and dysuria were monitored for timely treatment and recovery. They were followed up for four months to evaluate clinical effects of the combined therapy and its safety.^8^,^9^

### Statistical analysis

All data were analyzed by SPSS 17.0. The numerical data were compared with t test, and the categorical data were compared by using Chi-square test. P<0.05 was considered statistically significant.

## RESULTS

### Clinical treatment outcomes

**Fig.1:**
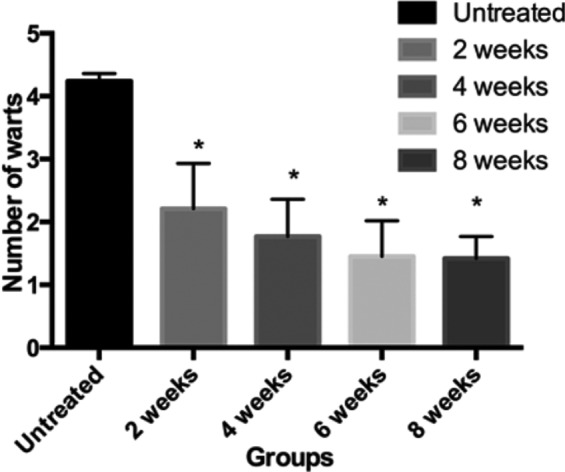
Numbers of warts at different time points vs. untreated group, *P<0.05.

**Fig.2:**
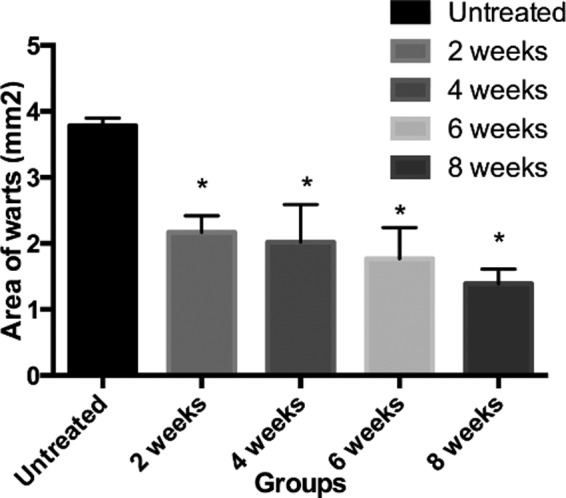
Area of warts at different time points vs. untreated group, *P<0.05.

Genital warts were mitigated in 107 out of the 110 cases (cure rate: 97.3%). After being treated once, 49 cases (44.5%) were cured, being males all. Then 28 (48.3%) and 21 (70.0%) cases were cured after being further treated once and twice respectively. The results are summarized in Table-I. Male patients had significantly better treatment outcomes at the urethral orifice than those in other affected parts. After treatment, the number and area of warts all decreased in postoperative 2nd, 4th, 6th and 8th weeks (Fig. 1 and 2).

**Table-I:** Clinical treatment outcomes of ALA-PDT (n=110, %).

Number of treatments	Number of cases	Cured	Partly cured	Ineffective
1	110	49 (44.5%)	58 (52.7%)	3 (2.7%)
2	58	28 (48.3%)	30 (51.7%)	0
3	30	21 (70.0%)	9 (30.0%)	0
4	9	5 (55.6%)	4 (44.4%)	0
>4	4	4 (100.0%)	0	0

### Relationship between gender and clinical therapeutic effects

In the 107 patients, the cure rate of male patients was 98.8%, and they were cured after being treated four times. In contrast, female patients, who were cured after five times of treatment, had the cure rate of 91.7%. Their cure rates were similar (χ^2^=0, P>0.05), but the males were cured significantly less times than the females (t=-7.432, P<0.05) (Table II and III).

**Table-II:** Clinical treatment outcomes of ALA-PDT for male patients (n=84, %).

Number of treatments	Number of cases	Cured	Partly cured	Ineffective
1	84	49 (58.3%)	34 (40.5%)	1 (1.2%)
2	34	22 (64.7%)	12 (35.3%)	0
3	12	9 (75.0%)	3 (25.0%)	0
4	3	3 (100.0%)	0	0

**Table-III:** Clinical treatment outcomes of ALA-PDT for female patients (n=26, %).

Number of treatments	Number of cases	Cured	Partly cured	Ineffective
1	26	0 (0.0%)	24 (92.3%)	2 (2.7%)
2	24	7 (29.2%)	17 (70.8%)	0
3	17	7 (41.2%)	10 (58.8%)	0
4	10	6 (60.0%)	4 (40.0%)	0
5	4	4 (100.0%)	0	0

### Adverse reactions

All patients were free from systemic adverse reactions. For the cured cases, warts regressed within five days and the injured parts fully recovered within 10 days. Five patients suffered from mild tingling or burning sensation upon dressing at the urethral orifice, and most cases felt similarly after being illuminated for five minutes, suggesting that they tolerated the combined therapy well. After illumination, the affected parts of a small portion of the patients were mildly red, swelling or painful, accompanied by mild edema that basically disappeared within three days. During follow-up, the injured parts suffered from scars, ulcers or redness and swelling.

### Recurrence

During follow-up, three patients were subjected to recurrence at the urethral orifice and then cured by ALA-PDT after further treatment for five weeks.

## DISCUSSION

In clinical practice, genital warts are routinely treated by laser, freezing, electrocautery, surgery or topical use of corrosive, cytotoxic drugs, but they often fail to meet anticipated requirement. Meanwhile, adverse reactions such as local ulcers, erosions or bleeding occur.^10^ In case of large areas, warts are prone to recurrence due to difficulty in radical treatment. Genital warts are mainly caused by subclinical infection of HPV. Given skin damages in special positions, such as those at the urethral orifice, genital warts cannot be cured with traditional strategies that only remove visible surface ones. Recurrence easily leads to urethral stricture by forming scars, thus burdening patients both economically and psychologically and evidently affecting the quality of life.^11^

ALA-PDT, as a novel method for treating genital warts, enjoys advantages such as convenience, efficiency and low recurrence rate.^12^-^14^ Cytotoxic chemical substances (e.g. reactive oxygen species such as singlet oxygen) are produced by excitation of photosensitive substances (e.g. porphyrin precursors) in affected tissues at specific wavelengths and frequencies. As a result, biomacromolecules in these tissues are damaged to cure the warts.

Male patients usually have affected parts at the glans penis, penis body, foreskin interior, coronal sulcus or perianal region, while female patients are affected at the vaginal orifice, labia majora, labia minora or vaginal vestibule. ALA-PDT functions mainly by directly destroying genital warts, by blocking microvessels in tissues surrounding the warts to accelerate depletion of oxygen and nutritional substances, as well as by exerting immune effects.^15^ After ALA-PDT, the patients were free from systemic adverse reactions and only subjected to local ulcers, erosions, redness, swelling, scars or painful urination. Moreover, the mild symptoms were alleviated without special medical treatment.^16^ The cure rate in this study was 97.3%, and male patients had significantly better treatment outcomes at the urethral orifice than those in other affected parts. As to the cured cases, genital warts regressed within five days and the injured parts fully recovered within 10 days. Five patients suffered from mild tingling or burning sensation upon dressing at the urethral orifice, and most cases felt similarly after being illuminated for five minutes, suggesting that they tolerated the combined therapy well. After illumination, the affected parts of a small portion of the patients were mildly red, swelling or painful, accompanied by mild edema that basically disappeared within three days. During the 4-month follow-up after treatment, the warts of three patients relapsed at the urethral orifice (recurrence rate: 2.7%). The recurrence may be attributed to insufficient supply of drugs or illumination time and dose that failed to penetrate tissues with pathological changes. Hence, photosensitive substances in wart tissues were hardly excited, augmenting the recurrence rate by preventing cells from necrosis or skin damages from pigmentation and cornification.

In summary, ALA-PDT is an efficient targeted therapy with high cure rate, low recurrence rate and safety. This novel method is particularly suitable for males at the urethral orifice, thus being worthy of promotion and application in clinical practice with first priority.
